# Community adaptation strategies toward tidal flood: A Case study in Langsa, Indonesia

**DOI:** 10.4102/jamba.v14i1.1258

**Published:** 2022-12-05

**Authors:** Furqan I. Aksa, Ramdan Afrian

**Affiliations:** 1 Department of Geography Education, Faculty of Education, University Samudra, Langsa, Indonesia

**Keywords:** hazard, disaster, community responses, tidal floods, adaption

## Abstract

Flood risk reduction in Indonesia is primarily still focused on structural measures with limited research assessing community adaptation strategies. The response and adaptation strategies carried out by the community have been recognized as playing an important role in planning future mitigation actions. Community adaptation strategies are key factors in disaster risk reduction. This study aimed to determine the responses and adaptation strategies to manage tidal floods by the coastal community of Langsa City. An in-depth interview with village heads and officials, youth leaders and the community was performed to generate data. The results showed that the local community ignored the dangers of tidal floods and disregarded them as life-threatening. Furthermore, they carried out adaptations, such as raising the house level using wood materials and building dams along the coastline autonomously without government intervention. The adaptations were carried out through communal work referred to as ‘gotong royong’ (mutual cooperation).

**Contribution:**

These findings highlight the importance of ‘gotong royong’ as social capital in disaster risk reduction and government attention to conduct integrated coastal area management.

## Introduction

According to the World Disaster Report data released in 2016, floods are the top global disaster occurrence at 43%, causing huge fatalities and economic losses. For instance, the floods in the South Asian region in 2017 killed 1200 people, while Europe experiences annual economic losses estimated at 4.3 billion euros (Roder, Hudson & Tarolli 2019). In 2019, Indonesia experienced 1312 floods incidents, which caused 367 deaths, 1385 injuries and affecting 649 659 people (Badan Nasional Penanggulangan Bencana [BNPB] 2019). Additionally, floods are predicted to increase in future as a result of climate change, population growth and changes in land use (Bergsma 2019). Over the past century, the Earth’s surface temperature has increased by 0.040 °C – 0.810 °C (Monirul Qader Mirza 2002), leading to higher sea levels, extreme rainfall and wind speed.

Indonesia is an archipelagic country with 81 000 km of coastline and more than 17 500 islands and therefore faces problems related to river floods, coastal inundation, displacement of lowlands and increasing water salinity (Marfai, Sekaranom & Ward 2015). Langsa City on the east coast of Aceh Province is one of Indonesia’s urban lowland and coastal communities in the region are threatened by floods, caused by increased tidal floods frequency because of climate change and sea-level rise. Tidal floods are a threat to urban development, affecting infrastructure and community settlements. This observation is supported by studies stating that increased residential buildings in flood hazard areas have increased flood disaster losses (Marfai et al. 2008, 2015). For instance, Langsa City has several slum settlements close to the coastal area because of cheap land prices and easy access by the fishermen, which make them vulnerable to flood risk.

Local communities have developed small-scale adaptation strategies to deal with disasters, based on their past experiences with the flood (Aksa 2021). These strategies have implications for community resilience (Nelson 2011). Vulnerability is conceptualised as a function of three elements such as exposure, sensitivity and adaptive capacity (Adger 2006). Adaptation is a conservative process focusing on human well-being maintenance or improvement in a particular socio-ecological system (Aksa et al. 2020; Nelson 2011). The adaptation strategies by traditional communities play an important role in flood risk management (Elrick-Barr et al. 2016) because they are the first responders to a disaster in their area. However, several studies such as Marfai et al. (2008, 2015), Marfai and Hizbaron (2011), related to the community adaptation strategies only focus on the coastal areas of Java Island, whereas there are no available studies in the coastal area of Langsa City, Aceh Province. Moreover, the flood hazard analysis by the National Disaster Management Agency (Badan Nasional Penanggulangan Bencana, [Indonesian National Board for Disaster Management]) in 2015 recorded a high flood hazard index in the area (BNPB 2015).

The flood risk reduction in Langsa City only focused on improving drainage and the community strategies did not get any attention. However, the local community’s coping capacity and adaptation for flood risk management were recognised (Grothmann & Reusswig 2006; Wisner et al. 2004). Similar studies should be conducted on the East coast of Aceh Province. This study therefore, aimed to identify the local communities’ responses and adaptation strategies in the coastal area of Langsa City to reduce the flood risk. Marfai and Hizbaron (2011) identified community response and adaptive capacity as a key role for disaster management and risk reduction programmes. The identified local knowledge can be practised in other coastal areas to reduce flood risk.

## Theoretical framework

### The concept of adaptation

Capacity is defined as a system’s ability to respond to change through learning, risk management, impact, new knowledge accumulation and effective management plans development (Ferro-Azcona et al. 2019; Masselink & Lazarus 2019). This study described adaptive capacity as a certain behaviour in which the system responds dynamically to changes (Lambeth 2016; Nelson 2011). In the context of climate change, adaptation is the process where individuals and communities reduce vulnerability and increase capacity in dealing with these changes (Ferro-Azcona et al. 2019; Harwitasari & Ast 2011; Marfai & Hizbaron 2011). Meanwhile, Intergovernmental Panel on Climate Change (IPCC), 2007 stated that adaptation is a necessary strategy at all stages and scales to complete climate change mitigation. Climate change adaptation is generally motivated by private and public interests (Harwitasari & Ast 2011). Satterthwaite et al. (2007) stated that adaptive capacity to climate change can be measured by density level, property ownership and knowledge. Harwitasari and Ast (2011) stated that climate change adaptation measures at the household level include elevating the house, raising the floor and yard level and building dams to prevent water.

The International Union for Conservation of Nature and Natural Resources (IUCN 2009) developed climate change social adaptations. This study only focused on adaptive capacity; however, there are five aspects in assessing community adaptation to climate change which include: vulnerability, exposure, social sensitivity, individual and community adaptive capacity. According to studies such as Marfai and King (2008), Marfai and Hizbaron (2011), Marfai et al. (2015), some indicators to assess adaptation strategies undertaken to counter coastal hazards include: raising house properties, increasing the floor level above the water level, increasing the yard level (in surrounding the house) and constructing a dam to block water from entering the house. This study followed the above indicators to assess the communities’ adaptation strategies in Langsa City.

### Tidal flood

Marfai and King (2008) stated that tidal floods are caused by high tide and accelerated sea-level rise. Global climate change can cause the frequency of tidal flood events to increase (Gobo, Amangabara & Pepple 2013), which affects communities’ social and economic conditions. According to Hinton (2000), the tidal flood hazard in coastal areas consists of coastal sedimentation and mixing of fresh as well as saltwater when tidal levels flow into rivers or other watercourses. Furthermore, tidal floods have indirect impacts such as disruption of public services (communication, electricity and clean water) and increased infectious diseases such as malaria (Marfai & King 2008).

### Study area

**FIGURE 1:**
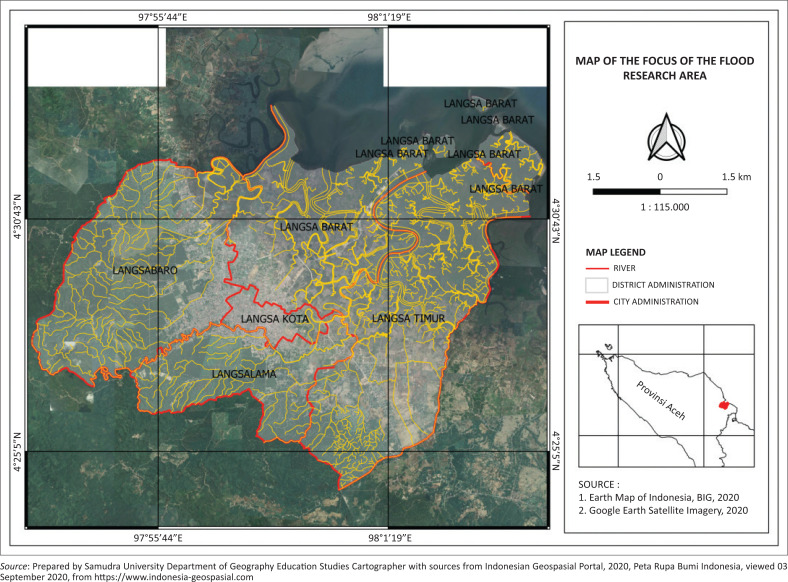
Study location.

This study was conducted in Indonesia’s Langsa City, Aceh Province, which has an alluvial lowland topography and an average rainfall of 10.85 mm per day (Jumiartanti 2020). It is located approximately 400 km from Banda Aceh City and consists of five sub-districts, namely, East Langsa, West Langsa, Langsa Kota, Langsa Baro and Langsa Lama (Figure 1). It is at an altitude of 0 m – 25 m above sea level, and most of the southwest area is a coastal alluvial lowland elevated at 8 m above sea level. The city’s southern part is a medium wavy fold mountain range elevated at about 75 m above sea level, whereas the eastern part is sedimentary swamps with a fairly wide distribution.

The coastal area consists of alluvial deposits with two micro areas, namely, alluvial deposits and tidal mangrove forests combined with alluvial deposits and mud, and ports and tourism. Most of the coastal communities live in the West Langsa sub-district directly adjacent to the Malacca Strait.

### Method

**FIGURE 2:**
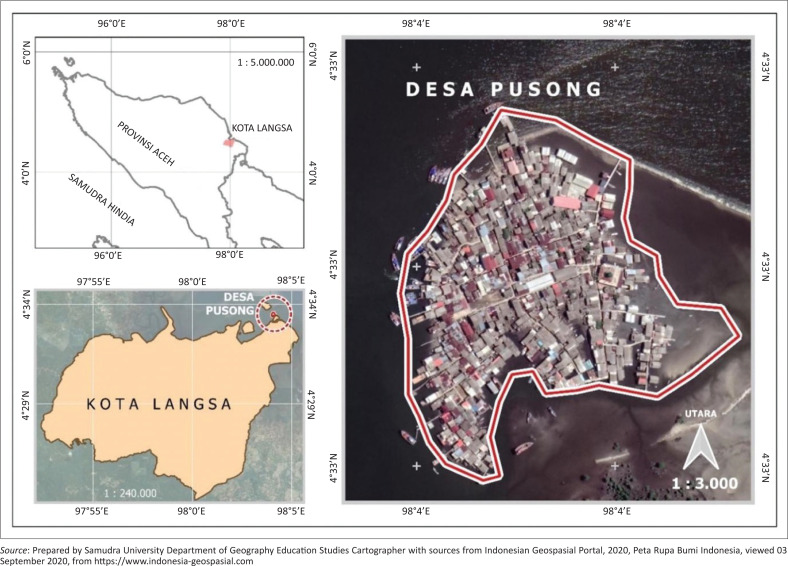
Pusong Village, West Langsa Sub-district.

This study used in-depth interviews and observations made by the Langsa City community living in flood risk areas to assess their responses and adaptation strategies to the hazard of tidal floods. Field observations showed that the Pusong Village, West Langsa Sub-district (Figure 2) had the highest tidal flood risk index in Langsa City.

Pusong Village is located in the West Langsa Sub-district and measures 250 ha with a population of 1797 (Central Bureau of Statistics 2019). The village was formed because of the community’s fishing activities in the Telaga Tujuh area, consisting of four hamlets, including Damai, Santosa, Sejahtera and Aman. The geographical conditions are unfavourable because of being in the middle of a small island regularly hit by tidal waves, hence experiencing tidal floods.

This study used community participatory methods derived from social inductive research and produced descriptive results (Marfai et al. 2015). Kumar (2002) pointed out that community participation methods can be built from interviews, observations, Venn diagrams and decision trees.

The participatory method consisted of several stages, such as observing, listening and asking questions to the local community (Marfai et al. 2015). This study used an unstructured questionnaire for in-depth interviews with the main content developed from Marfai et al. (2015). Specifically, the questionnaire tried to identify a series of tidal flood events in the area to determine their impact and how the community handled them. The information collected consisted of community perceptions about tidal floods dangers, the factors causing tidal floods in the future and adaptation strategies by the community to reduce the impact or damage. The identification of events after the floods allows the determination of the event chronology in the community history and helps anticipate future events.

**FIGURE 3:**
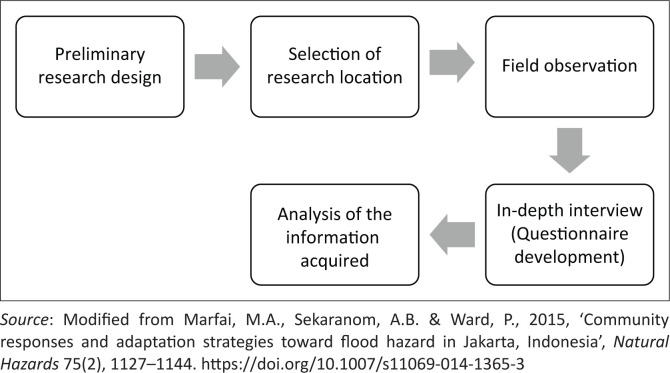
The method applied in this study.

Figure 3 shows all the stages used in the study.

The study used 50 participants represented by village heads, youth leaders, village officials and the community. The in-depth interview focused on obtaining information about the adaptation strategies carried out by the local community in reducing the tidal flood risk. The questionnaire consisted of the following components, depth and duration of inundation, tangible and intangible impacts of inundation, community perceptions of tidal floods hazard and adaptation strategies undertaken by the local community. Data were then analysed using descriptive statistics, such as simple percentages and summary tables.

### Ethical considerations

This article followed all ethical standards for research without direct contact with human or animal subjects.

## Results and discussion

### Socio-environmental condition of Pusong related to floods

**FIGURE 4:**
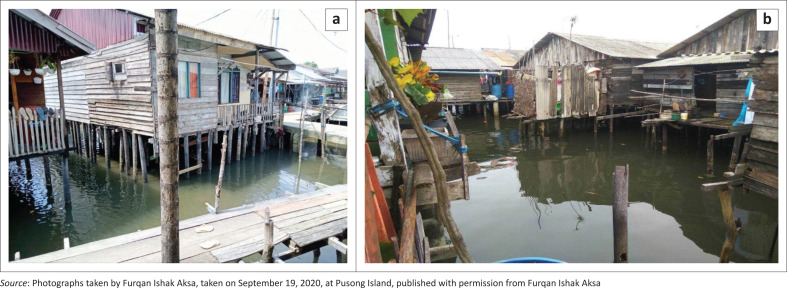
(a) The physical condition of building structures in Pusong Village; (b) Condition of community houses in Pusong Village.

Observations in the Pusong coastal area showed that the village is vulnerable to tidal floods caused by the physical (building) and social conditions of the community. The physical vulnerability is caused by the flood hazard area’s location and the use of wood for the main structure easily damaged by floods (Figure 4a, b). The type of building material used is an indicator of physical vulnerability (Arif, Mardiatna & Giyarsih 2017).

Furthermore, the area has limited infrastructure such as drainage, clean water and garbage disposal sites which causes a high cost of living and economic dependence on the mainland (Syamsidik et al. 2020). The unfavourable geographical conditions increase community vulnerability to coastal floods hazard and is unsuitable for settlement.

**FIGURE 5:**
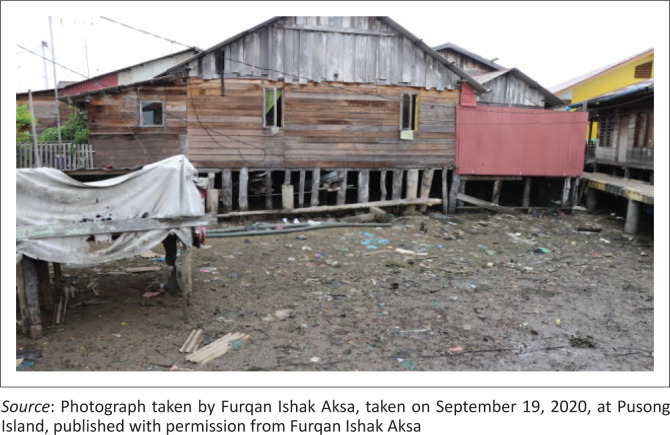
Environmental conditions in Pusong Village.

The area consists of slum settlements as shown by Mirza, Caisarina and Solehati (2018) that related to the slums rejuvenation model in Telaga Tujuh Village. The results showed that 365 houses in Pusong Village lacked clean water. The number of slum settlements increases the vulnerability of the community to coastal floods dangers and can cause damage. Some studies, such as Wiratuningsih, Setyowati and Suhandini (2018), stated that population density and the number of slum settlements can increase the community vulnerability to the tidal floods hazard. Furthermore, Pusong Village had bad environmental conditions that could cause a decline in the health level of the local community. Garbage swept during the high tide was found under residents’ houses, contributing to longer water inundation, which is common in coastal areas (Figure 5).

In-depth interviews showed that the residents in densely populated areas were mainly fishermen, sellers of daily necessities and lower-class workers, who originally came to find fish in the ‘Telaga Tujuh’ area but stayed because they got used to and comfortable with tidal floods. The village head stated in the interview that the Langsa City Government had relocated and provided simple houses to accommodate the community of Pusong Village. However, the community felt comfortable and preferred to live in the village because it is close to their work. This finding was similar to the study conducted by Bachri et al. (2015) which found that most people prefer to live in areas with high hazards because they are comfortable and have what they need.

**TABLE 1:** Community perception of tidal flood hazard.

No	Indicator	Question	Number of respondents	Percentage
1	Knowledge of tidal flood hazard	I am aware that Pusong Village experiences tidal floods regularly.	40	80
		I did not realize Pusong Village was experiencing tidal floods regularly.	20	10
2	Motivating the community to continue living in Pusong Village	I have no other choice but to stay in Pusong Village	35	70
		Accessibility to the city centre, industrial areas, public facilities and close to their workplace	42	84
		Pusong Village is my birthplace	32	64
3	The depth of tidal floods that is common in Pusong Village	< 25 cm	40	80
		25 cm – 50 cm	5	10
		50 cm – 75 cm	3	6
		75–100	2	4
		> 100	-	-
4	The duration of the tidal flood	< 3 h	43	86
		3–6 h	8	16
		6–9 h	-	-
		> 9 h	-	-
5	Causes of inundation	Land subsidence	18	36
		Sea level rise	15	30
		Do not know	25	50

### Community perception of tidal flood hazard

The local community realised the hazard of tidal floods but remained in the area with 70% claiming they had no other choice but to live in the area, 84% said the area was close to the city centre and their workplace, and 64% stated that Pusong Village was their birthplace. These findings concur with previous studies that stated high knowledge of hazards does not always support preparedness actions (Lambeth 2016).

In general, the findings indicated that the community’s risk perception of flood hazards tends to be high. In all, 80% of the respondents realised that Pusong Village regularly experienced floods but it was not enough reason for them to leave. This finding reinforced the result of the previous study that the perception of high risk does not always make people prepare for natural hazards (Wachinger et al. 2013). For example, most residents stayed because of kinship factors, as evidenced by in-depth community interviews. This is similar to Marfai and Hizbaron (2011) as well as Marfai and Hizbaron (2011) who examined the adaptation strategies of coastal communities in Terboyo Wetan Village and Trimulyo Village along the Semarang City coastline, showing the community stayed regardless of being aware of tidal floods dangers. This is because the community has long lived in the area and is close to their work as fishermen.

The study showed that 80% of Pusong Villagers said the tidal flood inundation in the area was > 25 cm and lasted more than 3 h, 50% did not know the cause, while 36% stated that it was caused by land subsidence, and 30% said it was caused by rising sea levels. The lack of public knowledge about inundation and causes of floods is attributed to poor education as well as non-involvement by Langsa City Government, non-governmental organisations (NGOs) and universities in increasing community capacity in the form of socialising the dangers of tidal floods. Dissemination of the dangers of coastal floods increases public knowledge about these hazards (Gaillard & Mercer 2013). The interview results also found that it is rare for local governments like the Regional Disaster Management Agency (BPBD) of Langsa City and universities to carry out programmes to reduce community capacity building in villages against the danger of tidal floods. Local government shows less attention to small islands regarding disaster risk reduction (Syamsidik et al. 2020).

### Community responses towards tidal floods

Findings showed that the tidal floods did not interfere with the community’s daily activities. All the respondents continued with their daily activities, such as washing (as shown in Table 2). This is irrational behaviour in protecting oneself from the danger of floods (Baker 2007).

**TABLE 2:** The impact of tidal floods on community activities.

Daily activities	Number of respondents		Percentage	
	Yes	No	Yes	No
Continuing daily activities (undisturbed by tidal floods)	36	14	72	28
Continuing household work				
Washing	50		100	-
Cooking	46		92	8

**TABLE 3:** Physical adaptations by respondents.

Physical adaptation by respondents	Number of respondents		Percentage	
	Yes	No	Yes	No
Raise the house	24	26	48	52
Raising the floor surface above the water level	20	30	40	60
Raising the level of the house (two floors)	12	38	24	76
Make a small dam to prevent water from entering the house	39	11	78	22

Respondents said that they continued to work as usual despite the high tide that inundated their homes, this shows that the community is used to the floods. Society has relied on several methods of adaptation to deal with flood danger as discussed below.

### Community adaptation strategies

In general, the adaptation strategies by coastal communities against the danger of tidal floods are done independently without the Langsa City government’s intervention. The survey showed that the physical adaptation strategies are strongly influenced by the economic conditions of the community. The strategies rely on several techniques, 40% raised the floor surface above the water level and elevated the house, while 24% raised the level of the house to two floors. This is presumably because it is expensive to raise the floor of the house and those who raised their houses to two floors were from high-producing households. This finding corroborated with the results of the study conducted by Elrick-Barr et al. 2016 which showed the adaptive capacity of the community depends on financial capital, social conditions and nature.

**FIGURE 6:**
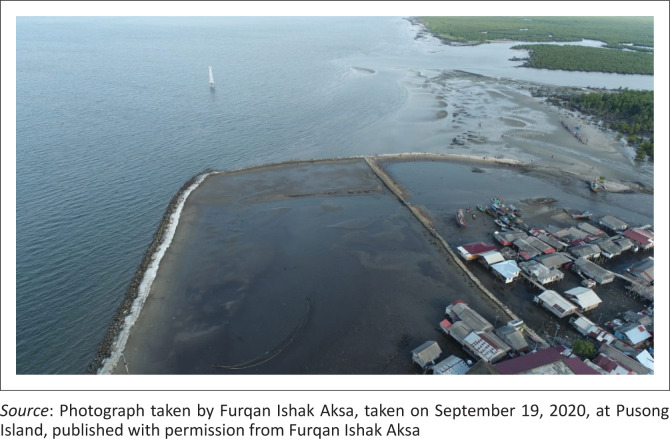
Construction of dams along the coastline.

**FIGURE 7:**
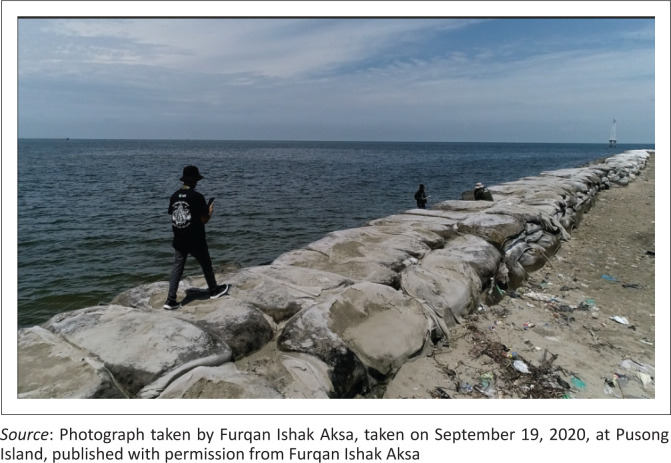
Embankment construction along the coastline.

**FIGURE 8:**
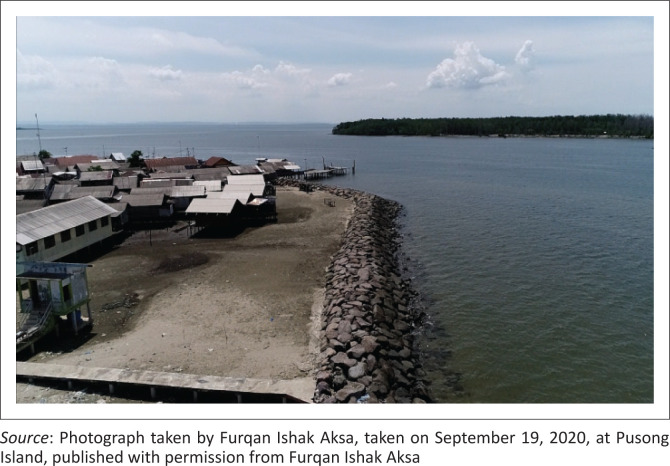
Embankments built along the coastline.

According to the village officials, communities independently built dams along the coastline (Figure 6, Figure 7 and Figure 8), following a cooperative system. This communal work system of ‘mutual cooperation’ is practised through non-physical adaptation strategies, mainly used after floods and include the reuse of undamaged building materials like wood. The community also applied the principle of ‘mutual cooperation’ to provide loans to relatives or neighbours to repair their houses that were affected by tidal floods and thus shortening the reconstruction period.

The communal work system known as ‘mutual cooperation’ reduces vulnerability and increases community capacity to flood risk, while promoting community inclusion in disaster response enabling them to act according to their capabilities (Taylor & Peace 2015). Mutual cooperation is considered as social capital that contributes to disaster risk reduction in Indonesia and can connect community networks to be shared with other network members (Kusumasari & Alam 2012).

Building dams along the area coastline can reduce the tidal discharge entering community settlements. Construction of dams started in 2018 to prevent puddles of water to flow inside people’s homes during high tide.

Findings suggested that most people still used autonomic adaptation. There are two types of adaptation strategies namely, autonomous and planned (Harwitasari & Ast 2011). Autonomous adaptation means actions that are not managed (reactive) without the government’s intervention, while planned adaptation is part of the government’s response strategy (Harwitasari & Ast 2011).

The government can adopt policies to reduce the vulnerability of the community to tidal floods danger in Langsa City. This can be conducted through integrated coastal area management policy per Law No. 27 of 2007 on coastal areas and small islands management. Article 23 paragraph 2 states that small islands and surrounding waters are prioritised for conservation, marine cultivation, tourism and the fishing industry. Furthermore, the government can determine the coastal border which is the land along the coast with a width of at least 100 m from the highest point of the tide (presidential regulation no. 51 of 2016). These policies are important because the coastal area has a complex geomorphological process that includes physical processes such as waves, tidal inundation, sea-level rise, erosion, sedimentation and various weather phenomena (Marfai et al. 2008). Natural geomorphic processes are a hazard for coastal areas residents and therefore, integrated coastal area management should be adopted on the coast of Langsa City, especially Pusong Village.

Well-managed adaptation strategies should be immediately implemented because the probability of coastal tidal floods is expected to increase in the future. IPCC (2007) predicted that the Indonesian sea level will rise 100 cm because of increased seawater temperature from 1.31 °C to 4.61 °C in 2100 (Harwitasari & Ast 2011).

## Conclusion

This study shows that the community has independent adaptation strategies for finding solutions to reduce the risk of coastal disasters. It disregards tidal floods that regularly occur in the area as a risk and ignores the danger. The adaptation capacity carried is on a small scale because of economic conditions. Specifically, the community depended on self-help funds to build dams and raise the floors of their houses. Therefore, there is a need for a better institutional adaptation capacity to the danger of tidal floods including in Langsa City. The government at Langsa City should plan clear strategic action plans to reduce the risk of tidal floods. Activities such as tidal flood disaster risk reduction forums and coastal flood’s danger awareness should continue.
